# National Institutes of Health Funding for Tuberculosis Comorbidities Is Disproportionate to Their Epidemiologic Impact

**DOI:** 10.1093/ofid/ofad618

**Published:** 2023-12-18

**Authors:** Madeline Carwile, Susie Jiaxing Pan, Victoria Zhang, Madolyn Dauphinais, Chelsie Cintron, David Flynn, Alice M Tang, Pranay Sinha

**Affiliations:** Section of Infectious Diseases, Department of Medicine, Boston University Chobanian & Avedisian School of Medicine, Boston, Massachusetts, USA; College of Arts and Sciences, Boston University, Boston, Massachusetts, USA; School of Arts and Sciences, Tufts University, Medford, Massachusetts, USA; Section of Infectious Diseases, Boston Medical Center, Boston, Massachusetts, USA; Section of Infectious Diseases, Boston Medical Center, Boston, Massachusetts, USA; Department of Medical Sciences and Education, Boston University Chobanian & Avedisian School of Medicine, Boston, Massachusetts, USA; Department of Public Health and Community Medicine, Tufts University School of Medicine, Boston, Massachusetts, USA; Section of Infectious Diseases, Department of Medicine, Boston University Chobanian & Avedisian School of Medicine, Boston, Massachusetts, USA; Section of Infectious Diseases, Boston Medical Center, Boston, Massachusetts, USA

**Keywords:** comorbidities, funding, HIV, tuberculosis, undernutrition

## Abstract

Tuberculosis (TB) is a leading infectious killer worldwide. We systematically searched the National Institutes of Health Research, Portfolio Online Reporting Tools Expenditures and Results (RePORTER) website to compare research funding for key TB comorbidities—undernutrition, alcohol use, human immunodeficiency virus, tobacco use, and diabetes—and found a large mismatch between the population attributable fraction of these risk factors and the funding allocated to them.

Tuberculosis (TB) has regained its position as the leading infectious killer worldwide, with 10.6 million new cases and 1.3 million deaths in 2022 [1]. Globally, the leading risk factors for TB disease are undernutrition, alcohol use, human immunodeficiency virus (HIV), smoking/tobacco use, and diabetes [1]. These comorbidities also affect the severity of a patient's TB disease, their likelihood of being cured of active disease, and mortality rates [1, 2].

One way to consider the impact of a risk factor is to measure its population attributable fraction (PAF). Per the World Health Organization (WHO), the PAF represents the “proportional reduction in population disease or mortality [that] would occur if exposure to a risk factor were reduced to an alternative ideal exposure scenario” [3]. In 2019, prior to the onset of the coronavirus disease 2019 (COVID-19) pandemic, the WHO estimated that the global PAFs for the key risk factors of TB incidence were as follows: undernourishment, 19%; alcohol use disorders, 8.1%; HIV infection, 7.7%; smoking, 7.1%; and diabetes, 3.1% [4]. The WHO uses a variety of data to calculate these figures, including data from the WHO and the World Bank, as well as scholarly papers from researchers who have studied these TB risk factors [1, 4, 5]. These PAFs can help guide public health officials and assist global health leaders in prioritizing action on comorbidities and should also be considered by funding agencies as they decide which research areas to support.

In this analysis, we assessed National Institutes of Health (NIH) funding for TB comorbidities to determine to what extent research funding correlates with the PAFs of these 5 critical TB comorbidities.

## METHODS

Three analysts (M. C., V. Z., and S. J. P.) systematically searched the NIH RePORTER website [6], with tie-breaking votes determined by a senior analyst (P. S.). Supplementary Table 1 shows the analysts for each search. Prior to beginning searches and analyses, the analysts (M. C. and V. Z.) discussed search terms with guidance and review by a research librarian (D. F.). Table 1 shows the search terms used.

**Table 1.** Search Terms, Initial Results, and Relevant Results for Each Search

Comorbidity	Search Terms	Results	2018–2019	2014	2009
Undernutrition	(Tuberculoses OR Tuberculosis OR TB) AND (Malnutrition OR “Nutritional Deficiency” OR “Nutritional Deficiencies” OR Undernutrition OR Malnourishment OR Malnourishments OR “Nutrition Disorder” OR “Nutritional Disorders” OR “Nutritional Disorder”)	Initial results	22	14	22
		Relevant results	4	2	4
Alcohol use	(Tuberculoses OR Tuberculosis OR TB) AND (“Alcohol Drinking” OR “Alcohol Drinking/adverse effects” OR “Alcohol Consumption” OR “Alcohol Intake” OR “Alcohol Drinking Habits” OR “Alcohol Drinking Habit” OR “Alcoholism” OR “Alcohol Abuse” OR “Alcohol”)	Initial results	57	20	26
		Relevant results	11	1	2
HIV	(Tuberculoses OR Tuberculosis OR TB) AND (HIV OR “Human Immunodeficiency Virus” OR “Acquired Immunodeficiency Syndrome” OR “AIDS Virus” OR “Acquired Immune Deficiency Syndrome Virus” OR “Acquired Immunodeficiency Syndrome Virus” OR AIDS)	Initial results	938	450	441
		Relevant results	175	64	52
Tobacco use	(Tuberculoses OR Tuberculosis OR TB) AND (Smoking OR “Smoking Behaviors” OR “Smoking Behavior” OR “Smoking Habit” OR “Smoking Habits” OR “Smoking/adverse effects” OR “Tobacco Smoking” OR “Cigar Smoking” OR “Cigarette Smoking”)	Initial results	27	16	9
		Relevant results	3	0	0
Diabetes	(Tuberculoses OR Tuberculosis OR TB) AND (“Diabetes Mellitus” OR Diabetes)	Initial results	89	36	37
		Relevant results	14	7	2

Abbreviations: HIV, human immunodeficiency virus; TB, tuberculosis.

The initial search was conducted for the years 2018–2019 (the 2 full years immediately prior to the start of the COVID-19 pandemic) and without enabling “Active Projects” (meaning that both active and inactive studies could be included). While the PAFs for each risk factor are usually similar from year to year, the 2019 PAFs were used to allow the best comparison with 2018–2019 data. To help reduce the likelihood that the observed results were due to short-term funding trends or an anomalous funding year, 1 analyst (M. C.) also reviewed the years 2014 and 2009. These years were selected as they allowed for an overview of results over a 10-year period, with 3 searches spaced approximately 5 years apart.

**Figure 1.**
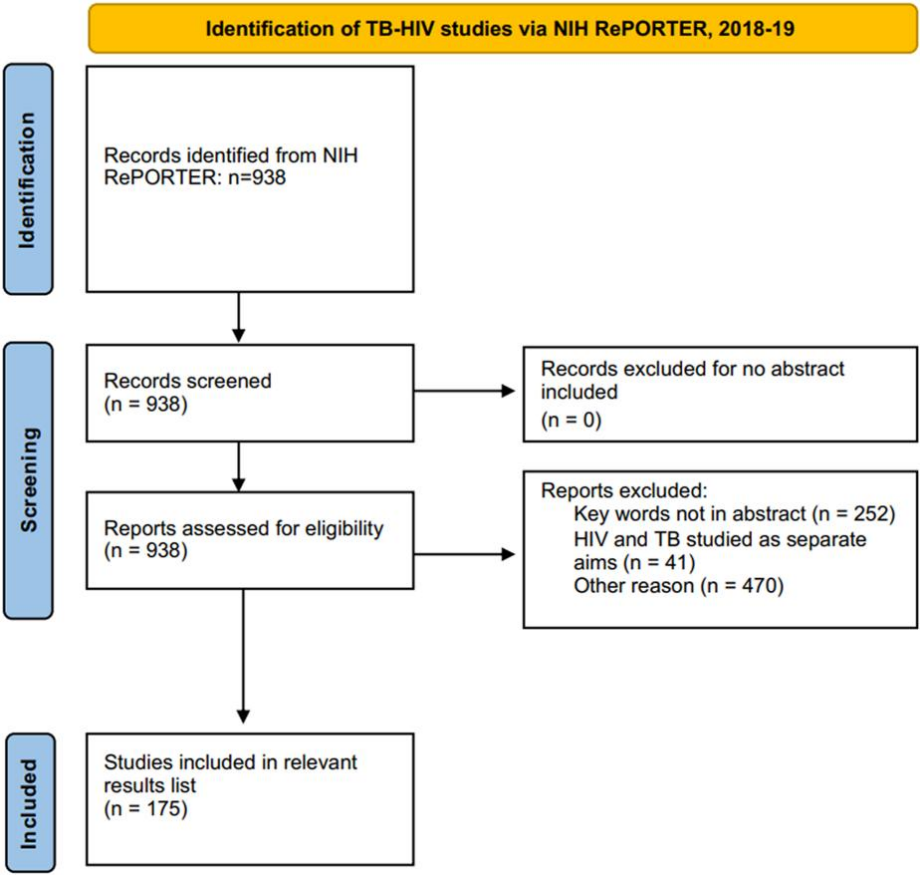
Preferred Reporting Items for Systematic Reviews and Meta-Analyses (PRISMA) diagram of tuberculosis (TB)–human immunodeficiency virus (HIV) studies identified via National Institutes of Health (NIH) Research, Portfolio Online Reporting Tools Expenditures and Results (RePORTER) for the 2018–2019 search. This figure uses the Prisma Flow Diagram from Macquarie University, available at https://libguides.mq.edu.au/systematic_reviews/prisma_screen.

For each study included in the initial search results, the analysts read the title and abstract to determine the study's relevance. The analysts included grants funding basic science, animal models, clinical research, translational work, and training, provided that the funding focused on the connection between TB and the risk factors of interest: undernutrition, alcohol use, HIV, tobacco use, and diabetes. In general, inclusion required that TB and the comorbidity be studied under the same aim and not as separate topics within the same grant. The analysts did include studies with up to 3 different aims or aspects provided that a substantial focus was on studying the relationship between TB and the specific comorbidity. When there was uncertainty, the analysts discussed the study with the senior analyst to determine eligibility. Studies were excluded if they did not include an abstract (unless the same project was listed in another year with the abstract included) or did not include keywords for both TB and the relevant comorbidity in the abstract itself. Figure 1 provides a visual representation of this process, with the number of studies identified, screened, excluded, and included for the 2018–2019 TB-HIV search.

The analysts recorded the study name, category (eg, training program, basic science, clinical research), a brief description of the study, the fiscal year, and funding (including direct and indirect costs). After conducting separate searches and analyses, the analysts met to discuss and compare their results and generated a final list of relevant studies with their total funding. Studies that looked at >1 risk factor (such as both undernutrition and HIV) were included in >1 list.

## RESULTS

### Studies Identified

The main analysis (2018–2019) initially identified 22 studies for undernutrition, 57 for alcohol use, 938 for HIV, 27 for tobacco use, and 89 for diabetes (Table 1). After reviewing the abstracts, the analysts identified the following numbers of relevant studies: 4 for undernutrition, 11 for alcohol use, 175 for HIV, 3 for tobacco use, and 14 for diabetes. Similarly, the number of HIV-TB studies far exceeded the number of funded studies for other TB comorbidities in our searches of 2009 and 2014 (Table 1). For example, HIV-TB studies had between 52 and 64 relevant search results in these 2 searches, while TB-undernutrition had between 2 and 4 relevant search results.

## Supplementary Material

ofad618_Supplementary_Data

## Figures and Tables

**Table 2.** Funding for Each Search, Compared to the Population Attributable Fraction

Comorbidity	Funding 2009	Funding 2014	Funding 2018–2019	Population Attributable Fraction (2019)
Undernutrition	$1.4 million	$1.2 million	$1.2 million	19.0%
	*$2.8 million*	*$2.4 million*		
Alcohol use	$582 000	$455 000	$5 million	8.1%
	*$1.2 million*	*$910 000*		
HIV	$14.2 million^a^	$24.6 million^b^	$87.6 million	7.7%
	*$28.4 million*	*$49.2 million*		
Tobacco use	$0	$0	$192 000	7.1%
	*$0*	*$0*		
Diabetes	$619 000	$2.5 million	$6 million	3.1%
	*$1.2 million*	*$5 million*		

For direct comparison with 2018–2019 funding levels, italicized figures represent the yearly funding doubled, that is, if 2 years were included instead of only 1 year. Funding levels are shown as United States dollars.

Abbreviation: HIV, human immunodeficiency virus.

^a^Twenty-seven studies were excluded as they did not have an abstract.

^b^Forty-five studies were excluded as they did not have an abstract.

## DISCUSSION

We believe that this brief report provides the first systematic analysis of NIH funding for key TB comorbidities. Through this systematic search of NIH-funded projects, we were able to quantify the differences in funding for research on principal comorbidities that drive the TB pandemic. We found that the NIH funding for TB risk factors was not proportional to the PAF of each risk factor. For example, although undernutrition has a global PAF of 19% to HIV's 7.7%, in 2018–2019 it only received $1.2 million in funding compared to $87.6 million for HIV. In all 3 searches, TB-HIV consistently received a substantially higher amount of funding compared to the other TB comorbidities. Even with a United States–centric view, the funding seems disproportionate as diabetes, tobacco use, and alcohol use also received lower funding compared to HIV-TB. In the United States, alcohol and tobacco use have a higher PAF as compared to undernutrition, HIV, and diabetes [7].

This analysis had some limitations. The analysis was limited to only the text included in the abstract, which may underestimate smaller projects outside of the scope of their abstract. For instance, larger HIV studies may also provide some funding for HIV-TB research, but may not state this due to the limited length of their abstract. This limitation may apply more to larger funding projects with multiple aims, and as such may actually underestimate the amount of funding provided for HIV-TB studies, which tended to have more aims and larger study focuses compared to the other search terms.

For many of the searches, there were substantially more initial results compared to the number of relevant results. This finding was likely due to the inclusion of Project Terms, which are keywords associated with the project. For instance, a substantial number of projects were tagged with “Malnutrition” but did not mention malnutrition in the abstract. Some studies exceeded 100 Project Terms, suggesting that not all may be relevant. We excluded studies where the comorbidity was listed in the Project Terms but not mentioned in the abstract.

Moreover, in studies with multiple aims, this analysis does not measure the amount of funding provided for each aim. For instance, in an HIV study where HIV-TB coinfection is one of multiple aims, the total amount of funding was listed with no knowledge of what percentage (eg, 10% or 90%) of the total funding went toward HIV-TB research specifically. This limitation may have overestimated the amount of funding for HIV-TB studies, as these studies were more likely to include multiple aims.

We acknowledge that a PAF is decidedly not the sole factor that should determine funding priority, but the discordance between the PAFs for TB comorbidities and available funding is striking. We do not recommend reallocation of funding from HIV to other comorbidities as this would constitute a zero-sum game that would ultimately decelerate progress on TB elimination. Instead, these results should prompt the NIH and other funders to consider providing additional funding for studies that investigate non–HIV-TB risk factors. Our analysis cannot discern whether differences in funding are driven by reduced scholarly interest or tepid interest from funders, or both. However, these identified gaps in funding for some key TB comorbidities can be readily rectified by issuing requests for applications and notifications of funding opportunities. Doing so could expedite the elimination of the leading infectious killer worldwide through science and discovery.

## Supplementary Data

Supplementary materials are available at *Open Forum Infectious Diseases* online. Consisting of data provided by the authors to benefit the reader, the posted materials are not copyedited and are the sole responsibility of the authors, so questions or comments should be addressed to the corresponding author.

## Notes

***Previous presentations.*** This work was presented at the American Thoracic Society Conference, Washington, District of Columbia, 21 May 2023.

***Patient consent.*** This study does not include factors necessitating patient consent.

***Disclaimer.*** The funders had no role in study design or manuscript writing.

***Financial support.*** This work was supported by the Warren Alpert Foundation (grant number 6005415) to P. S.; the Burroughs Wellcome Fund/American Society for Tropical Medicine and Hygiene (100000861 and 100001949) postdoctoral fellowship to P. S.; the National Institute of Allergy and Infectious Diseases (grant number 5T32 AI-052074-13 and K01AI167733-01A1 to P. S.); and the Civilian Research and Development Foundation (grant number DAA3-19-65673-1 and 100004419), as well as federal funds from the government of India's Department of Biotechnology (501100001407) and the Indian Council of Medical Research.
