# Supplementary material for: National Institutes of Health Funding for Tuberculosis Comorbidities Is Disproportionate to Their Epidemiologic Impact
Source: Open Forum Infect Dis. 2023 Dec 18;11(1):ofad618. doi: 10.1093/ofid/ofad618 (PMC10785210; doi:10.1093/ofid/ofad618)
Supplement: ofad618_Supplementary_Data [file ofad618_supplementary_data.docx]

**Supplemental Tables**

Supplemental Table 1: Analysts for each search

| **Search** | **Analyst(s)** |
| --- | --- |
| 2018-19 | MC, SP, and VZ |
| 2014 | MC |
| 2009 | MC |

Supplemental Table 2. List of studies with description, 2018-19

| Undernutrition | Role of nuclear receptors in nutritional and metabolic risk for tuberculosis | This study assesses the role of nuclear receptors and the impact of impaired vitamin A availability on immune response, control of bacterial growth, and TB disease outcome. | $114,000 |
| --- | --- | --- | --- |
| Undernutrition | Modulation of immunity to tuberculosis through vitamin A-dependent nuclear receptors | This study assesses the role of nuclear receptors and the impact of impaired vitamin A availability on immune response, control of bacterial growth, and TB disease outcome. | $190,000 |
| Undernutrition | Trial of Vitamin D Supplementation to Prevent TB Infection in Schoolchildren | This clinical trial examines whether vitamin D supplementation reduces the risk of acquisition of LTBI in children. | $673,745 |
| Undernutrition | Trial of Vitamin D Supplementation to Prevent TB Infection in Schoolchildren | This clinical trial examines whether vitamin D supplementation reduces the risk of acquisition of LTBI in children. | $200,000 |
| HIV | Computational and Molecular Epidemiology Training in TB and HIV in Uganda | This funding supports training in the molecular and computational epidemiology of TB and HIV, with a focus on the dynamics of TB/HIV coinfection. | $299,433 |
| HIV | Computational and Molecular Epidemiology Training in TB and HIV in Uganda | This funding supports training in the molecular and computational epidemiology of TB and HIV, with a focus on the dynamics of TB/HIV coinfection. | $297,482 |
| HIV | Training Program in Tuberculosis and HIV Research in Ghana | This program focuses on training researchers and strengthening institutional capacity in TB and HIV, with a particular focus in the area of TB/HIV coinfection. | $298,356 |
| HIV | Training Program in Tuberculosis and HIV Research in Ghana | This program focuses on training researchers and strengthening institutional capacity in TB and HIV, with a particular focus in the area of TB/HIV coinfection. | $297,034 |
| HIV | Dartmouth-Boston University HIV-TB Research Training for the Infectious Disease Institute at Muhimbili University of Health and Allied Sciences | This program focuses on research training in infectious diseases, including a focus on HIV/TB coinfection. | $299,614 |
| HIV | Dartmouth-Boston University HIV-TB Research Training for the Infectious Disease Institute at Muhimbili University of Health and Allied Sciences | This program focuses on research training in infectious diseases, including a focus on HIV/TB coinfection. | $303,688 |
| HIV | SWG1: HIV/TB Scientific Working Group | This funding will support an HIV/TB Scientific Working Group. | $34,397 |
| HIV | SWG1: HIV/TB Scientific Working Group | This funding will support an HIV/TB Scientific Working Group. | $34,245 |
| HIV | Research Training Program in the Diagnosis, Treatment, and Prevention of AIDS-related MDR-TB in Haiti | This funding will provide two years of research training to 10 Haitian clinicians with a primary focus on HIV/TB coinfection and AIDS-related MDR-TB. | $2,000 |
| HIV | Research Training Program in the Diagnosis, Treatment, and Prevention of AIDS-related MDR-TB in Haiti | This funding will provide two years of research training to 10 Haitian clinicians with a primary focus on HIV/TB coinfection and AIDS-related MDR-TB. | $290,429 |
| HIV | A Nurse Case Management Intervention to Improve MDR-TB/HIV Coinfection Outcomes | This funding supports a cluster randomized study to determine the impact and cost-effectiveness of a nurse case management intervention on MDR-TB treatment for MDR-TB/HIV coinfected patients. | $602,190 |
| HIV | A Nurse Case Management Intervention to Improve MDR-TB/HIV Coinfection Outcomes | This funding supports a cluster randomized study to determine the impact and cost-effectiveness of a nurse case management intervention on MDR-TB treatment for MDR-TB/HIV coinfected patients. | No funding stated. |
| HIV | Clinical Phenotyping: Pediatric HIV and TB Cohort | This funding supports the Collaborative African Genomics Network by funding topics in HIV, TB, and HIV/TB coinfection. | $163,041 |
| HIV | Clinical Phenotyping: Pediatric HIV and TB Cohort | This funding supports the Collaborative African Genomics Network by funding topics in HIV, TB, and HIV/TB coinfection. | $232,868 |
| HIV | CAfGEN Administrative Centre | This funding supports the Collaborative African Genomics Network by funding topics in HIV, TB, and HIV/TB coinfection. | $743,827 |
| HIV | CAfGEN Administrative Centre | This funding supports the Collaborative African Genomics Network by funding topics in HIV, TB, and HIV/TB coinfection. | $237,346 |
| HIV | Molecular mechanisms of TB exposure outcomes among HIV-infected children | This funding supports the Collaborative African Genomics Network to examine host genetics of HIV and TB disease progression, including a focus on TB disease progression among HIV-infected children. | $187,905 |
| HIV | Molecular mechanisms of TB exposure outcomes among HIV-infected children | This funding supports the Collaborative African Genomics Network to examine host genetics of HIV and TB disease progression, including a focus on TB disease progression among HIV-infected children. | $219,140 |
| HIV | Role of Inducible Bronchus Associated Lymphoid Tissue in Latent Tuberculosis | This study uses mouse and nonhuman primate models to study several topics in Mtb infection and progression to pulmonary TB in TB/HIV coinfection. | $800,095 |
| HIV | Role of Inducible Bronchus Associated Lymphoid Tissue in Latent Tuberculosis | This study uses mouse and nonhuman primate models to study several topics in Mtb infection and progression to pulmonary TB in TB/HIV coinfection. | $800,058 |
| HIV | Novel Methods to Inform HIV/TB Clinical Trial Development | This funding covers a variety of clinical trials in the field of HIV/TB coinfection. | $745,684 |
| HIV | Novel Methods to Inform HIV/TB Clinical Trial Development | This funding covers a variety of clinical trials in the field of HIV/TB coinfection. | $732,206 |
| HIV | Pharmacokinetic and Programmatic Evaluations to Optimize HIV/TB Co-Treatment Regimens in Children | This study assesses pediatric HIV/TB co-treatment options by examining 10 years of treatment data from over 5500 pediatric patients enrolled in the Harvard/AIDS Prevention Initiative in Nigeria. | $198,504 |
| HIV | Pharmacokinetic and Programmatic Evaluations to Optimize HIV/TB Co-Treatment Regimens in Children | This study assesses pediatric HIV/TB co-treatment options by examining 10 years of treatment data from over 5500 pediatric patients enrolled in the Harvard/AIDS Prevention Initiative in Nigeria. | $198,932 |
| HIV | HIV-associated Tuberculosis Training Program (HATTP) | This funding will train researchers at the University of Cape Town in topics related to HIV/TB coinfection. | $307,666 |
| HIV | HIV-associated Tuberculosis Training Program (HATTP) | This funding will train researchers at the University of Cape Town in topics related to HIV/TB coinfection. | $311,783 |
| HIV | Impact of Once-Weekly Rifapentine and Isoniazid on the Steady State Pharmacokinetics of Dolutegravir and Darunavir Boosted with Cobicistat in Healthy Volunteers (R2D2) | This study uses healthy volunteers to test a treatment for LTBI in HIV-positive individuals. | None stated |
| HIV | Impact of Once-Weekly Rifapentine and Isoniazid on the Steady State Pharmacokinetics of Dolutegravir and Darunavir Boosted with Cobicistat in Healthy Volunteers (R2D2) | This study uses healthy volunteers to test a treatment for LTBI in HIV-positive individuals. | None stated |
| HIV | NanoDisk-MS measured Mtb antigen peptides for TB diagnosis and treatment monitoring in HIV-infected children | This study tests nanodisks that can detect Mtb-Ag in the blood of HIV-infected children to improve TB diagnosis in HIV-infected children. | $209,047 |
| HIV | How does HIV lead to increased susceptibility to tuberculosis? | This study uses cellular and molecular analysis to determine how HIV infection promotes the intracellular growth of Mtb and impacts the immune environment of the lung to encourage infection and progression of TB. | $546,571 |
| HIV | How does HIV lead to increased susceptibility to tuberculosis? | This study uses cellular and molecular analysis to determine how HIV infection promotes the intracellular growth of Mtb and impacts the immune environment of the lung to encourage infection and progression of TB. | $551,971 |
| HIV | Pitt HIV-TB research and training program in India | This funding supports training for healthcare professionals in the areas of HIV, TB, and HIV/TB co-infections. | $303,546 |
| HIV | Pitt HIV-TB research and training program in India | This funding supports training for healthcare professionals in the areas of HIV, TB, and HIV/TB co-infections. | $378,546 |
| HIV | Promoting Engagement in the Drug Resistant TB/HIV Care Continuum in South Africa | This funding aims to enhance adherence and retention in care for M/XDR- TB/HIV patients in South Africa via community adherence groups. | $516,583 |
| HIV | Promoting Engagement in the Drug Resistant TB/HIV Care Continuum in South Africa | This funding aims to enhance adherence and retention in care for M/XDR- TB/HIV patients in South Africa via community adherence groups. | $543,856 |
| HIV | Mechanisms of antigen-specific CD4 T cell dysfunction in HIV/TB co-infection | This study examines potential mechanisms of HIV-associated dysregulation of Mtb-specific CD4 T cell responses in individuals with HIV/TB co-infection. | $223,125 |
| HIV | Mechanisms of antigen-specific CD4 T cell dysfunction in HIV/TB co-infection | This study examines potential mechanisms of HIV-associated dysregulation of Mtb-specific CD4 T cell responses in individuals with HIV/TB co-infection. | $267,750 |
| HIV | Effect of HIV infection on M. tuberculosis granuloma formation and evolution | This study investigates the effects of HIV infection on Mtb granulomas, with the goal of designing therapeutics that more effectively target HIV and TB. | $292,500 |
| HIV | Advancing point-of-care diagnostics for integrated HIV and TB care in South Africa | This study evaluates a second-generation urinary LAM assay for HIV-associated TB and a clinic-based assay for HIV. | $72,811 |
| HIV | Individualized approaches to the HIV/TB epidemic among polysubstance users in Irkutsk | This study examines the influence of substance use (including heroin injection and alcohol use) patterns on adherence, pharmacokinetics and disease progression of HIV/TB in Irkutsk, Siberia. | $220,000 |
| HIV | Individualized approaches to the HIV/TB epidemic among polysubstance users in Irkutsk | This study examines the influence of substance use (including heroin injection and alcohol use) patterns on adherence, pharmacokinetics and disease progression of HIV/TB in Irkutsk, Siberia. | $180,000 |
| HIV | Rapid Immune Restoration and Lung Injury in HIV/TB | This prospective cohort study in South Africa examines the immunologic mechanisms associated with incident lung damage during TB treatment among people with HIV/TB coinfection. | $664,038 |
| HIV | Rapid Immune Restoration and Lung Injury in HIV/TB | This prospective cohort study in South Africa examines the immunologic mechanisms associated with incident lung damage during TB treatment among people with HIV/TB coinfection. | $714,852 |
| HIV | The effect of HIV exposure and infection on immunity to TB in children | This study uses longitudinal cohorts of infants and children in Kenya to assess the effect of maternal HIV exposure and infant HIV infection on induction of trained immunity to Mtb in BCG-vaccinated infants, and the capacity of ART to restore innate and adaptive anti-mycobacterial immune responses in HIV-infected children. | $828,368 |
| HIV | Impact of concurrent HIV and latent TB therapies on Mtb-specific immune function | This study uses a macaque aerosol model of LTBI and SIV coinfection to identify the components of TB immunity in the blood and lung compartments that remain impaired after ART. | $899,688 |
| HIV | Impact of concurrent HIV and latent TB therapies on Mtb-specific immune function | This study uses a macaque aerosol model of LTBI and SIV coinfection to identify the components of TB immunity in the blood and lung compartments that remain impaired after ART. | $1,034,989 |
| HIV | The Impact of Pre-exisiting SIV on Host Immunity to M tuberculosis in Macaques | This study uses a macaque model to identify the mechanism by which pre-existing SIV infection interferes with host control over an Mtb infection. | $740,051 |
| HIV | The Impact of Pre-exisiting SIV on Host Immunity to M tuberculosis in Macaques | This study uses a macaque model to identify the mechanism by which pre-existing SIV infection interferes with host control over an Mtb infection. | $751,157 |
| HIV | PREVINE-TB: Prevent: Evaluating the implementation of New strategies for preventive TB among people living with HIV in Brazil | This study examines ways to prevent TB among people living with HIV in Brazil, including tuberculin skin testing (TST) for latent TB infection (LTBI) and adherence to 6 months of isoniazid preventive therapy (IPT). | $574,554 |
| HIV | PREVINE-TB: Prevent: Evaluating the implementation of New strategies for preventive TB among people living with HIV in Brazil | This study examines ways to prevent TB among people living with HIV in Brazil, including tuberculin skin testing (TST) for latent TB infection (LTBI) and adherence to 6 months of isoniazid preventive therapy (IPT). | $669,384 |
| HIV | C-type Lectin Receptor Pathways in the Pathogenesis of TB/HIV Co-infection | This study aims to identify HIV-mediated defects and demonstrate the impact of these defects in the setting of pulmonary TB. | $750,254 |
| HIV | C-type Lectin Receptor Pathways in the Pathogenesis of TB/HIV Co-infection | This study aims to identify HIV-mediated defects and demonstrate the impact of these defects in the setting of pulmonary TB. | $748,275 |
| HIV | Host biomarkers for M. tuberculosis infection activity in HIV-infected persons | This study examines host protein and antibody responses as correlates for Mtb infection activity in asymptomatic people living with HIV. | $667,954 |
| HIV | Host biomarkers for M. tuberculosis infection activity in HIV-infected persons | This study examines host protein and antibody responses as correlates for Mtb infection activity in asymptomatic people living with HIV. | $399,489 |
| HIV | Host biomarkers for M. tuberculosis infection activity in HIV-infected persons | This study examines host protein and antibody responses as correlates for Mtb infection activity in asymptomatic people living with HIV. | $286,465 |
| HIV | Impact of a multimodal intervention to reduce dual stigma and improve treatment outcomes in HIV/Drug-resistant TB co-infected patients in KwaZulu-Natal, South Africa | This funding supports the study of an intervention to promote patient engagement in a continuum of M/XDR-TB/HIV care by addressing issues of stigma. | $186,251 |
| HIV | Impact of a multimodal intervention to reduce dual stigma and improve treatment outcomes in HIV/Drug-resistant TB co-infected patients in KwaZulu-Natal, South Africa | This funding supports the study of an intervention to promote patient engagement in a continuum of M/XDR-TB/HIV care by addressing issues of stigma. | $227,584 |
| HIV | Heme oxygenase-1 and the bioenergetic threshold of latent TB and HIV co-infection | This study examines the role of heme oxygenase-1 in TB/HIV coinfection. | $335,520 |
| HIV | Heme oxygenase-1 and the bioenergetic threshold of latent TB and HIV co-infection | This study examines the role of heme oxygenase-1 in TB/HIV coinfection. | $335,520 |
| HIV | Role of IL-17 Cytokine Networks in TB Relapse Due to HIV | This study uses a mouse model of HIV-mediated TB relapse to identify the mechanistic role of Th17 cells and IL-17 in TB containment, with the goal of developing preventive measures to reduce TB recurrence in those at risk due to HIV coinfection. | $554,261 |
| HIV | Quantitative diagnosis of TB/HIV co-infection using pathogen-specific exosomes in blood | This study develops and tests an assay to diagnose TB, HIV, and TB/HIV coinfection. | $75,750 |
| HIV | Quantitative diagnosis of TB/HIV co-infection using pathogen-specific exosomes in blood | This study develops and tests an assay to diagnose TB, HIV, and TB/HIV coinfection. | $78,208 |
| HIV | Autophagy Against Tuberculosis and HIV | This study examines the role of autophagy and associated processes in active TB, TB latency, and HIV/TB interactions. | $744,529 |
| HIV | Autophagy Against Tuberculosis and HIV | This study examines the role of autophagy and associated processes in active TB, TB latency, and HIV/TB interactions. | $658,214 |
| HIV | Pharmacokinetics of Anti-tuberculosis and Antiretroviral Drugs in Children | This study assesses the pharmacokinetics of pediatric anti-TB and antiretroviral drugs, including a focus on children with TB/HIV coinfection. | $623,600 |
| HIV | Pharmacokinetics of Anti-tuberculosis and Antiretroviral Drugs in Children | This study assesses the pharmacokinetics of pediatric anti-TB and antiretroviral drugs, including a focus on children with TB/HIV coinfection. | $660,869 |
| HIV | Effects of HIV SIV on unconventional T cells in immunity to M. tuberculosis in pre adolescents | This study uses a macaque model of SIV/TB to model HIV/Mtb coinfected children and determine whether a preexisting SIV infection impairs T cells. | $1,301,851 |
| HIV | Predictors of treatment toxicity, failure, and relapse in HIV-related tuberculosis | This study enrolls 800 Brazilian participants to understand the relationship between human genetic single nucleotide polymorphisms, TB and HIV drug levels, and TB treatment outcomes, with the goal of improving treatment for HIV-related TB. | $641,929 |
| HIV | Predictors of treatment toxicity, failure, and relapse in HIV-related tuberculosis | This study enrolls 800 Brazilian participants to understand the relationship between human genetic single nucleotide polymorphisms, TB and HIV drug levels, and TB treatment outcomes, with the goal of improving treatment for HIV-related TB. | $648,910 |
| HIV | Host susceptibility to Mycobacterium tuberculosis recurrence in HIV-infected people | This funding supports training for a researcher studying topics related to recurrence of TB in people infected with HIV. | $192,780 |
| HIV | Host susceptibility to Mycobacterium tuberculosis recurrence in HIV-infected people | This funding supports training for a researcher studying topics related to recurrence of TB in people infected with HIV. | $192,780 |
| HIV | Safety, pharmacokinetics, and resistance to bedaquiline in XDR TB and HIV | This study examines bedaquiline, a TB drug, among XDR and pre-XDR TB patients, including those who are receiving ART for HIV. | $734,343 |
| HIV | Safety, pharmacokinetics, and resistance to bedaquiline in XDR TB and HIV | This study examines bedaquiline, a TB drug, among XDR and pre-XDR TB patients, including those who are receiving ART for HIV. | $547,193 |
| HIV | Safety, pharmacokinetics, and resistance to bedaquiline in XDR TB and HIV | This study examines bedaquiline, a TB drug, among XDR and pre-XDR TB patients, including those who are receiving ART for HIV. | $168,228 |
| HIV | Dolutegravir for the treatment of HIV in patients with Tuberculosis in KwaZulu-Natal South Africa | This study examines the impact of dolutegravir-based ART regimens on HIV treatment outcomes among patients who have HIV/TB coinfection and are being treated for TB. | $102,834 |
| HIV | Modeling approaches to prioritize TB prevention among people with HIV in Uganda | This funding supports training for a researcher interested in modeling preventive therapy for TB prevention in HIV-infected individuals. | $129,222 |
| HIV | Host-Directed Therapy to Augment anti-M. tuberculosis Responses in the Setting of HIV Co-infection and to Sterilize the Tuberculoma | This study uses a macaque model to examine the role of IDO (an immunosuppressant of activated CD4+ T cells) in the reactivation of HIV/SIV in individuals with LTBI. | $916,047 |
| HIV | Host-Directed Therapy to Augment anti-M. tuberculosis Responses in the Setting of HIV Co-infection and to Sterilize the Tuberculoma | This study uses a macaque model to examine the role of IDO (an immunosuppressant of activated CD4+ T cells) in the reactivation of HIV/SIV in individuals with LTBI. | $866,688 |
| HIV | Host-Directed Therapy to Augment anti-M. tuberculosis Responses in the Setting of HIV Co-infection and to Sterilize the Tuberculoma | This study uses a macaque model to examine the role of IDO (an immunosuppressant of activated CD4+ T cells) in the reactivation of HIV/SIV in individuals with LTBI. | $6,506 |
| HIV | Point-of-care C-reactive protein-based tuberculosis screening in people living with HIV: a randomized trial | This trial evaluates the impact of a TB screening strategy in individuals with HIV, with a focus on clinical outcomes and cost-effectiveness. | $963,941 |
| HIV | URBAN ARCH (3/5) Uganda Cohort TB preventive therapy for HIV-infected alcohol users in Uganda: an evaluation of safety tolerability and adherence | This study assesses the safety and tolerability of TB preventive therapy for HIV-infected alcohol drinkers, and examines if the benefits in preventing TB outweigh the risks of hepatoxicity associated with heavy alcohol use. | $538,240 |
| HIV | URBAN ARCH (3/5) Uganda Cohort TB preventive therapy for HIV-infected alcohol users in Uganda: an evaluation of safety tolerability and adherence | This study assesses the safety and tolerability of TB preventive therapy for HIV-infected alcohol drinkers, and examines if the benefits in preventing TB outweigh the risks of hepatoxicity associated with heavy alcohol use. | $536,867 |
| HIV | Novel clinic-based TB diagnostics and testing algorithms for persons with HIV | This study aims to develop an optimized clinical algorithm to identify HIV-infected outpatients for TB testing and to determine the diagnostic accuracy of tests to detect TB in HIV-infected patients in South Africa. | $188,985 |
| HIV | Impact of Weekly Administration of RPT and INH on TAF Pharmacokinetics in Healthy Volunteers | This study uses healthy volunteers to test the safety of a drug regimen for LTBI in HIV-positive individuals. | No funding stated. |
| HIV | Impact of Weekly Administration of RPT and INH on TAF Pharmacokinetics in Healthy Volunteers | This study uses healthy volunteers to test the safety of a drug regimen for LTBI in HIV-positive individuals. | No funding stated. |
| HIV | Novel Structure-Based Rifamycins for Drug-resistant TB and HIV Co-infection | This study examines candidates for drug development for HIV/TB coinfection, with a focus on targeting MTB RNA polymerase. | $641,007 |
| HIV | Novel Structure-Based Rifamycins for Drug-resistant TB and HIV Co-infection | This study examines candidates for drug development for HIV/TB coinfection, with a focus on targeting MTB RNA polymerase. | $641,007 |
| HIV | Immune correlates of tuberculosis and non-tuberculosis infectious morbidity in Southern African HIV-exposed, uninfected infants. | This study examines topics related to tuberculosis infection in HIV-exposed, uninfected infants. | $724,699 |
| HIV | Development of Gleevec for TB and TB/HIV | This study assesses the efficacy of Gleevec, a cancer drug, in a non-human primate model of infection with TB and TB/SIV. | $1,571,205 |
| HIV | Development of Gleevec for TB and TB/HIV | This study assesses the efficacy of Gleevec, a cancer drug, in a non-human primate model of infection with TB and TB/SIV. | $1,534,659 |
| HIV | Studies of Mucosal Associated Invariant T (MAIT) cells in people with HIV, TB and HIV/TB co-infection in South Africa | This funding supports a five-year research training plan for a scientist focusing on topics related to HIV, TB, and HIV/TB co-infection. | $193,482 |
| HIV | Studies of Mucosal Associated Invariant T (MAIT) cells in people with HIV, TB and HIV/TB co-infection in South Africa | This funding supports a five-year research training plan for a scientist focusing on topics related to HIV, TB, and HIV/TB co-infection. | $194,066 |
| HIV | Impact of tuberculosis on the development and function of the immune system in SIV-infected infants | This study uses a macaque model of SIV/TB to better understand the immunopathogenesis of TB in HIV-infected children. | $1,200,027 |
| HIV | Multidrug-resistant tuberculosis and HIV: Composition of Strains and biomarkers of treatment response | This funding supports a researcher’s training in several topics related to TB/HIV coinfection, including markers of TB treatment response and spatial diversity of Mtb strains within HIV-coinfected individuals. | $137,085 |
| HIV | Perturbation of antigen-specific T cell responses in latent TB/SIV co-infection | This study uses a macaque model to understand how HIV perturbs the latent control of Mtb infection. | $890,058 |
| HIV | Perturbation of antigen-specific T cell responses in latent TB/SIV co-infection | This study uses a macaque model to understand how HIV perturbs the latent control of Mtb infection. | $822,232 |
| HIV | Immune control mechanisms of TB latency in the setting of HIV co-infection | This study examines immunological and cellular mechanisms involved in host control of latent and active TB in the context of HIV infection. | $442,500 |
| HIV | Immune control mechanisms of TB latency in the setting of HIV co-infection | This study examines immunological and cellular mechanisms involved in host control of latent and active TB in the context of HIV infection. | $442,500 |
| HIV | Defining drivers of TB transmission in the era of universal ART, and implications for finding the walking well | This study assesses the contribution of people taking ART to TB transmission, with aims including comparing TB infectiousness of those taking ART to those not taking ART. | $498,771 |
| HIV | Human genetics of TB resistance in HIV-infected persons | This study examines the role of alveolar macrophages in TB resistance in HIV-infected persons to identify the molecular basis of Mtb infection. | $1,456,994 |
| HIV | Human genetics of TB resistance in HIV-infected persons | This study examines the role of alveolar macrophages in TB resistance in HIV-infected persons to identify the molecular basis of Mtb infection. | $1,693,450 |
| HIV | Effect of pregnancy and HIV on the development of tuberculosis | This study examines the impact of pregnancy on the host immune response to M. tuberculosis, with a focus on the development of active TB in postpartum HIV-infected women. | $192,780 |
| HIV | Effect of pregnancy and HIV on the development of tuberculosis | This study examines the impact of pregnancy on the host immune response to M. tuberculosis, with a focus on the development of active TB in postpartum HIV-infected women. | $192,780 |
| HIV | Provide Options for Treatment of Exposed Children against Tuberculosis (PROTECT) Study | This study assesses the attitudes of key stakeholders (caregivers, children, and healthcare providers) regarding TB preventive therapy for HIV+ and HIV- children. | $164,000 |
| HIV | Provide Options for Treatment of Exposed Children against Tuberculosis (PROTECT) Study | This study assesses the attitudes of key stakeholders (caregivers, children, and healthcare providers) regarding TB preventive therapy for HIV+ and HIV- children. | $198,320 |
| HIV | Influence of SIV replication on TB progression and immunity | This study uses a macaque model to characterize the influence of simian immunodeficiency (SIV) infection on Mtb infection, with the goal of understanding how HIV infection affects TB progression. | $1,029,305 |
| HIV | Influence of SIV replication on TB progression and immunity | This study uses a macaque model to characterize the influence of simian immunodeficiency (SIV) infection on Mtb infection, with the goal of understanding how HIV infection affects TB progression. | $933,104 |
| HIV | Clinical Pharmacology of Efavirenz Combined with High Dose Rifapentine | This funding supports career development for a researcher studying HIV and TB, with a particular focus on options for the treatment of latent and active TB in patients who are also infected with HIV. | $164,763 |
| HIV | Clinical Pharmacology of Efavirenz Combined with High Dose Rifapentine | This funding supports career development for a researcher studying HIV and TB, with a particular focus on options for the treatment of latent and active TB in patients who are also infected with HIV. | $164,973 |
| HIV | Impact of maternal HIV on Mycobacterium tuberculosis infection among peripartum women and their infants | This funding supports a researcher studying the relationship between maternal HIV and increased risk of Mtb infection in peripartum women and their infants. It will support a prospective cohort study examining the prevalence of Mtb infection in HIV-exposed and HIV-unexposed infants. | $184,588 |
| HIV | Impact of maternal HIV on Mycobacterium tuberculosis infection among peripartum women and their infants | This funding supports a researcher studying the relationship between maternal HIV and increased risk of Mtb infection in peripartum women and their infants. It will support a prospective cohort study examining the prevalence of Mtb infection in HIV-exposed and HIV-unexposed infants. | $184,588 |
| HIV | Defining the Impact of Immunodeficiency Virus Infection on Mycobacteria-Specific, Unconventional CD8+ T cells | This study uses a macaque model to study Mtb-specific CD8+ T cells with the goal of understanding how HIV dismantles immunity to TB. | $213,713 |
| HIV | Trial of Metformin for TB/HIV Host-directed Therapy | This study tests the anti-diabetic drug metformin as a treatment for TB in TB/HIV-coinfected patients. | $571,971 |
| HIV | Predictors of Resistance Emergence Evaluation in MDR-TB Patients on Treatment (PREEMPT) | This cohort study in India and Brazil follows 400 patients with MDR-TB over three years, with one aim being to see whether HIV seropositivity is a risk factor for low serum drug concentrations and/or the emergence of resistance. | $884,552 |
| HIV | Indoleamine dioxygenase suppresses pulmonary T-cell immunity to Mycobacterium tuberculosis | This study uses a macaque model of HIV/TB coinfection to assess how the expression of IDO, an immunosuppressant of activated CD4+ T cells, is increased in the lung granulomata of macaques. | $414,115 |
| HIV | Options for Delivery of Short-Course Tuberculosis Preventive Therapy: The 3HP Options Trial | This study uses a randomized control trial and economic modeling to assess a regimen of isoniazid and rifapentine, a treatment used for TB in people living with HIV. | $614,073 |
| HIV | Options for Delivery of Short-Course Tuberculosis Preventive Therapy: The 3HP Options Trial | This study uses a randomized control trial and economic modeling to assess a regimen of isoniazid and rifapentine, a treatment used for TB in people living with HIV. | $725,916 |
| HIV | HIV and Mycobacterial Disease in Mali | This funding supports developing training and research capacity in HIV and TB, including a strong focus on HIV infection and its mycobacterial comorbidities. | $303,095 |
| HIV | Epigenetic & Post-Translational Mechanisms of Macrophage Resistance to Mycobacterium tuberculosis During HIV Co-Infection | This funding supports epigenetic and proteomic studies to assess mechanisms of Mtb resistance in HIV+ individuals. | $1,043,172 |
| HIV | Epigenetic & Post-Translational Mechanisms of Macrophage Resistance to Mycobacterium tuberculosis During HIV Co-Infection | This funding supports epigenetic and proteomic studies to assess mechanisms of Mtb resistance in HIV+ individuals. | $1,077,894 |
| HIV | PpiA, a multitasking mycobacterial virulence protein | This study examines PpiA, a cyclophilin-like peptidyl-prolyl isomerase secreted by MTB, with a particular focus on how it may alter the host response during HIV/TB coinfection. | $245,625 |
| HIV | South African Medical Research Council Clinical Trials Unit (MRC CTU) | This funding supports clinical trials related to TB and HIV, with a focus on integrating TB and HIV care, and one aim focusing specially on HIV comorbidities, including TB. | $3,525,766 |
| HIV | South African Medical Research Council Clinical Trials Unit (MRC CTU) | This funding supports clinical trials related to TB and HIV, with a focus on integrating TB and HIV care, and one aim focusing specially on HIV comorbidities, including TB. | $1,084,670 |
| HIV | High-throughput identification of common CD8+ T cell responses to SIV and M. tuberculosis in rhesus macaques | This study uses a macaque model to assess CD8+ T cell responses to SIV and TB. | $684,547 |
| HIV | Resistance to MTB infection in HIV infected individuals in Uganda and S. Africa | This study examines HHCs in Uganda and miners in South Africa to understand why some HIV+ individuals resist latent Mtb infection, with a focus on transcriptional responses to Mtb in macrophages. | $2,270,572 |
| HIV | Resistance to MTB infection in HIV infected individuals in Uganda and S. Africa | This study examines HHCs in Uganda and miners in South Africa to understand why some HIV+ individuals resist latent Mtb infection, with a focus on transcriptional responses to Mtb in macrophages. | $2,422,204 |
| HIV | METABOLIC REPROGRAMMING OF T CELL ENERGY METABOLISM IN TUBERCULOSIS AND HIV | This study examines how Mtb/HIV reprograms immunometabolic pathways, and whether this can be reversed by host-directed therapy. The study will use human TB/HIV lung tissue and provide further information for TB/HIV vaccine design. | $510,497 |
| HIV | METABOLIC REPROGRAMMING OF T CELL ENERGY METABOLISM IN TUBERCULOSIS AND HIV | This study examines how Mtb/HIV reprograms immunometabolic pathways, and whether this can be reversed by host-directed therapy. The study will use human TB/HIV lung tissue and provide further information for TB/HIV vaccine design. | $522,518 |
| HIV | Pattern Recognition Receptors and Autophagy in Mtb Control in AIDS | This study examines how autophagy factors protect against Mtb infection, with a focus on HIV-Mtb co-pathogenesis. | $493,666 |
| HIV | Pattern Recognition Receptors and Autophagy in Mtb Control in AIDS | This study examines how autophagy factors protect against Mtb infection, with a focus on HIV-Mtb co-pathogenesis. | $492,962 |
| HIV | Interventions to reduce alcohol use and increase adherence to TB preventive therapy among HIV/TB co-infected drinkers (DIPT 2/2) | This trial of HIV/TB co-infected adults with heavy alcohol use examines ways to reduce alcohol use and increase adherence to treatment. | $525,645 |
| HIV | Interventions to reduce alcohol use and increase adherence to TB preventive therapy among HIV/TB co-infected drinkers (DIPT 2/2) | This trial of HIV/TB co-infected adults with heavy alcohol use examines ways to reduce alcohol use and increase adherence to treatment. | $531,617 |
| HIV | Interventions to reduce alcohol use and increase adherence to TB preventive therapy among HIV/TB co-infected drinkers (DIPT 1/2) | This trial of HIV/TB co-infected adults with heavy alcohol use examines ways to reduce alcohol use and increase adherence to treatment. | $516,226 |
| HIV | Interventions to reduce alcohol use and increase adherence to TB preventive therapy among HIV/TB co-infected drinkers (DIPT 1/2) | This trial of HIV/TB co-infected adults with heavy alcohol use examines ways to reduce alcohol use and increase adherence to treatment. | $518,556 |
| HIV | Immunotherapy Targeting MTB Persisters in the DC-impaired Setting of HIV and TB | This study tests a DNA vaccine targeting Mtb stringent response genes in a mouse model of TB, including mice who have a depletion of CD4+ T cells and persistent expansion of CD8+ T cells as occurs in HIV. | $204,688 |
| HIV | Immunotherapy Targeting MTB Persisters in the DC-impaired Setting of HIV and TB | This study tests a DNA vaccine targeting Mtb stringent response genes in a mouse model of TB, including mice who have a depletion of CD4+ T cells and persistent expansion of CD8+ T cells as occurs in HIV. | $245,563 |
| HIV | Impact of HIV infection on early responses to TB exposure | This funding supports career development for a clinical investigator studying various topics related to TB/HIV coinfection. | $191,484 |
| HIV | Impact of HIV infection on early responses to TB exposure | This funding supports career development for a clinical investigator studying various topics related to TB/HIV coinfection. | $193,484 |
| HIV | Capturing spatial patterns of new M. tuberculosis infection in Kampala, Uganda | This study assesses biomarkers for recent development of LTBI, with a focus on enrolling both HIV+ and HIV- adults. | $1,588,271 |
| HIV | Contribution of the CD153/CD30 axis to Mycobacterium tuberculosis control in humans | This study examines how Mtb-specific CD4+ T cells from individuals with LTBI express the molecule CD153, with one of the three main tasks being to define the impact of HIV infection on Mtb-specific CD4 T cells expressing CD153. | $176,702 |
| HIV | Childhood Tuberculosis Infection Among School-Age Children in Rural Uganda | This study examines several topics related to childhood TB infection, including the impact of perinatal HIV exposure on TB infection among HIV- uninfected school-age children. | $194,892 |
| HIV | Childhood Tuberculosis Infection Among School-Age Children in Rural Uganda | This study examines several topics related to childhood TB infection, including the impact of perinatal HIV exposure on TB infection among HIV- uninfected school-age children. | $194,386 |
| HIV | Pharmacokinetics and tolerability of adjunctive linezolid for the treatment of tuberculous meningitis | This clinical trial tests Rifampin and linezolid in patients who have TB meningitis and are coinfected with HIV. | $146,147 |
| HIV | Pharmacokinetics and tolerability of adjunctive linezolid for the treatment of tuberculous meningitis | This clinical trial tests Rifampin and linezolid in patients who have TB meningitis and are coinfected with HIV. | $176,179 |
| HIV | The role of antibodies in infant TB prevention | This study examines the role of antibodies in protection of HIV-exposed uninfected infants from Mtb infection. | $190,008 |
| HIV | Role of Lipid Antigen-Specific T Cells in the Anti-mycobacterial Immune Response of BCG/SIV Infected Macaques | This study uses a macaque model to assess the role of CD1-restricted immune responses in protection against TB and the impact of SIV infection on their anti-mycobacterial effector functions. | $212,500 |
| HIV | Role of Lipid Antigen-Specific T Cells in the Anti-mycobacterial Immune Response of BCG/SIV Infected Macaques | This study uses a macaque model to assess the role of CD1-restricted immune responses in protection against TB and the impact of SIV infection on their anti-mycobacterial effector functions. | $274,455 |
| HIV | Simplified Isoniazid Preventive Therapy (SPIRIT) Strategy to Reduce TB Burden | This study tests an intervention to increase the uptake of simplified INH preventive therapy, a TB treatment for HIV+ patients. | $683,851 |
| HIV | Simplified Isoniazid Preventive Therapy (SPIRIT) Strategy to Reduce TB Burden | This study tests an intervention to increase the uptake of simplified INH preventive therapy, a TB treatment for HIV+ patients. | $1,087,552 |
| HIV | Characterizing the pharmacokinetics of high dose rifampicin and linezolid in a randomized controlled trial for HIV-associated tuberculous meningitis | This RCT evaluates the safety of a treatment of rifampicin and linezolid in South Africans with HIV-associated TB meningitis. | $93,850 |
| HIV | Immune correlates of LTBI in HIV-exposed infants | This study analyzes immunity to TB in HIV exposed uninfected infants by profiling CD4 T cells and conducting other analyses. | $650,269 |
| HIV | TB and Other Pulmonary Complications of AIDS Research Training Program | This funding supports training for researchers and support personnel at Makerere University, Uganda, in the topics of TB and other pulmonary complications of HIV. | $300,208 |
| HIV | TB and Other Pulmonary Complications of AIDS Research Training Program | This funding supports training for researchers and support personnel at Makerere University, Uganda, in the topics of TB and other pulmonary complications of HIV. | $304,727 |
| HIV | GH16-005, Lesotho, Provide Miner-friendly SErvices for Integrated TB/HIV Care (PROMISE) | This prospective cohort study aims to evaluate the effectiveness, feasibility and acceptability of integrated TB/HIV services for migrant miners and their family members. | No funding stated. |
| HIV | GH16-005, Lesotho, Provide Miner-friendly SErvices for Integrated TB/HIV Care (PROMISE) | This prospective cohort study aims to evaluate the effectiveness, feasibility and acceptability of integrated TB/HIV services for migrant miners and their family members. | $462,449 |
| HIV | Anti-TB sterilizing immunity in immune and HIV+ individuals | This study assesses how HIV destroys protective mechanisms leading to enhanced TB susceptibility and severity and investigates the immune mechanisms of anti-TB immunity in HIV-infected individuals. | $672,467 |
| HIV | Research Training Program in the Diagnosis, Treatment, and Prevention of AIDS-related MDR-TB in Haiti | This funding supports two years of research training for 10 Haitian clinicians in a variety of topics related to AIDS-related TB. | $130,000 |
| HIV | Research Training Program in the Diagnosis, Treatment, and Prevention of AIDS-related MDR-TB in Haiti | This funding supports two years of research training for 10 Haitian clinicians in a variety of topics related to AIDS-related TB. | $364,464 |
| HIV | Research Training on Pathogenesis and Diagnosis of HIV-TB | This funding supports training and capacity building for researchers and clinicians in India on the subject of pulmonary and extrapulmonary TB in HIV+ patients. | $304,400 |
| HIV | HIV-induced transcriptional changes in alveolar macrophages in susceptibility to M. tuberculosis infection | This study examines how HIV infection increases risk of active TB infection by studying bronchoalveolar lavage fluid and assessing the impact of direct and indirect HIV infection on alveolar macrophages. | $49,524 |
| HIV | NK cell-mediated regulation of T cell immunity in TB/HIV co-infection | This study assesses the effect of HIV/TB coinfection on the mechanisms of natural killer cells and the regulation of memory T cell responses. | $688,115 |
| HIV | HIV-TB Co-infection: Tracking TB emergence after asymptomatic (latent) infection | This study uses a non-human primate model to understand TB relapse/reactivation in individuals coinfected with HIV/TB. | $615,075 |
| HIV | Targeted Immunotherapy for Tuberculosis and HIV co-infection | This study uses a mouse model to test the efficacy of diphtheria toxin-based cytokine receptor targeted fusion proteins in the selective elimination of cells associated with TB and HIV coinfection. | $204,558 |
| HIV | HIV Co-infections in Uganda: TB, Cryptococcus, and Viral Hepatitis. | This funding supports training for researchers in Uganda to study HIV coinfections. One of the three main areas in HIV/TB coinfection. | $292,517 |
| HIV | Optimal dosing of 1st line antituberculosis and antiretroviral drugs in children | This study assesses the pharmacokinetics of first line anti-TB drugs in children, with a particular focus on children with HIV who are also receiving antiretroviral drugs. | $36,396 |
| HIV | Transitioning from Pediatric to Adult HIV Care in Kenya | This study examines the cascade of care in TB among HIV-infected adolescents in Kenya using physical and electronic medical records. | $73,150 |
| HIV | Real-time Detection of Active TB in HIV Exposed Children on Customized Nanotrap | This funding assesses a diagnostic test for active TB in HIV-infected children. | $387,896 |
| HIV | Evaluation of novel tuberculosis screening strategies for people living with HIV | This funding supports a researcher studying topics in TB/HIV coinfection, including diagnostic strategies for TB among people living with HIV. | $197,640 |
| HIV | HIV-induced Defects in Pulmonary Macrophages Exacerbate Mycobacterium Tuberculosis Co-infection | This study uses a mouse model to understand how HIV infection disrupts the innate immune response of pulmonary macrophages to Mtb. | $705,641 |
| HIV | SOC Core | This funding supports access to genomics expertise and resources at five sites, with a focus on topics in HIV and TB/HIV coinfection. | $531,487 |
| HIV | HIV and Mycobacterial Disease in Mal | This funding will provide training and development support to researchers in Bamako, Mali, with a particular focus on HIV-associated mycobacterial infection, including HIV/TB. | $303,269 |
| HIV | Addiction, HIV and Tuberculosis in Malaysian Criminal Justice Settings | This study examines a variety of topics related to addiction, HIV, and TB among prisoners in Malaysia. Several aims focus specifically on TB prevention and treatment among HIV+ prisoners. | $656,887 |
| HIV | Rifampicin Malabsorption in HIV+ Patients: Mechanisms and Recovery after HAART | This funding supports training for a researcher studying the interaction between HIV infection and the absorption of rifampicin, a key anti-TB drug. | $166,104 |
| HIV | Point-of-care C-reactive protein-based tuberculosis screening in people living with HIV: a randomized trial | This funding evaluates the impact of a potentially more effective and cost-effective TB screening strategy for PLHIV | 963,941 |
| HIV | Patient-Oriented Research and Mentoring in HIV-associated Pulmonary Diseases | This funding supports a researcher's training in HIV-associated pulmonary diseases. While the aims focus on HIV with pneumonia and COPD, to study these aims the researcher will conduct a longitudinal study of 25 HIV-infected pneumonia patients and 25 HIV-infected TB patients. | $181,443 |
| Diabetes | Latent tuberculosis infection and risk of type 2 diabetes mellitus in US veterans | This study examines the relationship between latent TB infection and risk of diabetes mellitus by using longitudinal data from the Veterans Affairs Corporate Data Warehouse. | $77,568 |
| Diabetes | Latent tuberculosis infection and risk of type 2 diabetes mellitus in US veterans | This study examines the relationship between latent TB infection and risk of diabetes mellitus by using longitudinal data from the Veterans Affairs Corporate Data Warehouse. | $80,110 |
| Diabetes | The impact of diabetes on tuberculosis incidence and mortality in HIV-positive adults | This study examines the impact of type II diabetes on HIV treatment outcomes and TB risk, incidence, and mortality. | $44,465 |
| Diabetes | Impact of Diabetes and Hyperlipidemia on Host Defense | This study uses a mouse model to examine several research questions related to TB susceptibility in diabetes. | $418,750 |
| Diabetes | Impact of Diabetes and Hyperlipidemia on Host Defense | This study uses a mouse model to examine several research questions related to TB susceptibility in diabetes. | $418,750 |
| Diabetes | Altered immune-endocrine axis in type 2 diabetes and tuberculosis risk | This study assesses HHCs of TB cases in South Africa and Texas to examine the link between type II diabetes and risk of TB. | $336,930 |
| Diabetes | Altered immune-endocrine axis in type 2 diabetes and tuberculosis risk | This study assesses HHCs of TB cases in South Africa and Texas to examine the link between type II diabetes and risk of TB. | $340,941 |
| Diabetes | Urine Colorimetry for Tuberculosis Pharmacokinetics Evaluation in Children and Adults | This study assesses urine colorimetry to detect low anti-tuberculosis drug exposures and stratifies enrollment based on diabetes mellitus status in order to focus on adults with TB/diabetes coinfection. | $335,562 |
| Diabetes | Urine Colorimetry for Tuberculosis Pharmacokinetics Evaluation in Children and Adults | This study assesses urine colorimetry to detect low anti-tuberculosis drug exposures and stratifies enrollment based on diabetes mellitus status in order to focus on adults with TB/diabetes coinfection. | $458,304 |
| Diabetes | Urine Colorimetry for Tuberculosis Pharmacokinetics Evaluation in Children and Adults | This study assesses urine colorimetry to detect low anti-tuberculosis drug exposures and stratifies enrollment based on diabetes mellitus status in order to focus on adults with TB/diabetes coinfection. | $807,746 |
| Diabetes | India International Center for Excellence in Research | This study examines the role of pro-inflammatory cytokines in host immunity to TB, with a focus on TB patients who also have diabetes. | $1,917,556 |
| Diabetes | Glutathione depletion in lung parenchyma and its effects on Mtb infection | This study assesses how glutathione (GSH), a tripeptide elevated in patients with type II diabetes, affects immune defense against Mtb infection. The goal is to assess whether appropriate GSH-enhancing agents can be used to prevent the development of active TB in individuals with type II diabetes. | $423,000 |
| Diabetes | Research Training at the Confluence of Infectious and Non-Communicable Diseases in India | This funding supports research training in a variety of areas related to infectious and chronic diseases in India. TB and diabetes are specified as two major areas of study. | $216,284 |
| Diabetes | Mechanisms of Diabetic Susceptibility to Tuberculosis | This study uses diabetic guinea pigs to identify mechanisms of altered immune function in diabetes-tuberculosis comorbidity. | $123,093 |
| Alcohol use | The impact of alcohol consumption on TB treatment outcomes | This study recruits TB patients in South Africa in order to examine the associations between problem alcohol use and TB treatment outcomes. | $646,900 |
| Alcohol use | The impact of alcohol consumption on TB treatment outcomes | This study recruits TB patients in South Africa in order to examine the associations between problem alcohol use and TB treatment outcomes. | $646,074 |
| Alcohol use | Interventions to reduce alcohol use and increase adherence to TB preventive therapy among HIV/TB co-infected drinkers (DIPT 1/2) | This randomized controlled trial tests interventions for reduced drinking and increased medication adherence for HIV/TB co-infected drinkers in sub-Saharan Africa. | $148,569 |
| Alcohol use | Interventions to reduce alcohol use and increase adherence to TB preventive therapy among HIV/TB co-infected drinkers (DIPT 1/2) | This trial of HIV/TB co-infected adults with heavy alcohol use examines ways to reduce alcohol use and increase adherence to treatment. | $516,226 |
| Alcohol use | Interventions to reduce alcohol use and increase adherence to TB preventive therapy among HIV/TB co-infected drinkers (DIPT 1/2) | This trial of HIV/TB co-infected adults with heavy alcohol use examines ways to reduce alcohol use and increase adherence to treatment. | $518,556 |
| Alcohol use | Interventions to reduce alcohol use and increase adherence to TB preventive therapy among HIV/TB co-infected drinkers (DIPT 2/2) | This trial of HIV/TB co-infected adults with heavy alcohol use examines ways to reduce alcohol use and increase adherence to treatment. | $525,645 |
| Alcohol use | Interventions to reduce alcohol use and increase adherence to TB preventive therapy among HIV/TB co-infected drinkers (DIPT 2/2) | This trial of HIV/TB co-infected adults with heavy alcohol use examines ways to reduce alcohol use and increase adherence to treatment. | $531,617 |
| Alcohol use | URBAN ARCH (3/5) Uganda Cohort TB preventive therapy for HIV-infected alcohol users in Uganda: an evaluation of safety tolerability and adherence | This study assesses the safety and tolerability of TB preventive therapy for HIV-infected alcohol drinkers, and examine if the benefits in preventing TB outweigh the risks of hepatoxicity associated with heavy alcohol use. | $538,240 |
| Alcohol use | URBAN ARCH (3/5) Uganda Cohort TB preventive therapy for HIV-infected alcohol users in Uganda: an evaluation of safety tolerability and adherence | This study assesses the safety and tolerability of TB preventive therapy for HIV-infected alcohol drinkers, and examine if the benefits in preventing TB outweigh the risks of hepatoxicity associated with heavy alcohol use. | $536,867 |
| Alcohol use | Individualized approaches to the HIV/TB epidemic among polysubstance users in Irkutsk | This study examines the influence of substance use (including heroin injection and alcohol use) patterns on adherence, pharmacokinetics and disease progression of HIV/TB in Irkutsk, Siberia. | $220,000 |
| Alcohol use | Individualized approaches to the HIV/TB epidemic among polysubstance users in Irkutsk | This study examines the influence of substance use (including heroin injection and alcohol use) patterns on adherence, pharmacokinetics and disease progression of HIV/TB in Irkutsk, Siberia. | $180,000 |
| Tobacco use | Pulmonary impairment after tuberculosis in Georgia: Enhancing clinical research capacity to address the intersection of non-communicable diseases and tuberculosis | This study examines the extent to which MDR-TB, smoking, and biomarkers of lung inflammation contribute to increased prevalence of pulmonary impairment post-TB. | $192,100 |
| Tobacco use | How Does Nicotine Impair Macrophage Killing of Mycobacterium Tuberculosis? | This study uses an animal model with unexposed or nicotine- exposed mice, infects the recipient mice with MTB, and quantifies MTB burden, macrophage and T cell phenotypes, lung histopathology, and survival. | No funding stated. |
| Tobacco use | How Does Nicotine Impair Macrophage Killing of Mycobacterium Tuberculosis? | This study uses an animal model with unexposed or nicotine- exposed mice, infects the recipient mice with MTB, and quantifies MTB burden, macrophage and T cell phenotypes, lung histopathology, and survival. | No funding stated. |
